# Design, synthesis, and biological evaluation of novel derivatives of dithiodiglycolic acid prepared via oxidative coupling of thiols

**DOI:** 10.1080/14756366.2019.1575372

**Published:** 2019-02-12

**Authors:** Olga Bakulina, Anton Bannykh, Mirna Jovanović, Ilona Domračeva, Ana Podolski-Renić, Raivis Žalubovskis, Milica Pešić, Dmitry Dar’in, Mikhail Krasavin

**Affiliations:** a Saint Petersburg State University, Saint Petersburg, Russian Federation;; b Institute for Biological Research “Siniša Stanković”, University of Belgrade, Belgrade, Serbia;; c Latvian Institute of Organic Synthesis, Riga, Latvia;; d Faculty of Materials Science and Applied Chemistry, Institute of Technology of Organic Chemistry, Riga Technical University, Riga, Latvia

**Keywords:** TrxR, disulphide inhibitors, dithiodiglycolic acid, anticancer activity

## Abstract

Human thioredoxin reductase 1 (TrxR1) is a selenocysteine-containing enzyme which plays a crucial role in regulating numerous redox signalling pathways within the cell. While its functioning is important in all cells, levels of TrxR1 expression are higher in cancer cells, possibly as an adaptation to much higher levels of reactive oxygen species and the need for more extensive DNA synthesis. This makes TrxR1 an attractive target for cancer therapy development. Inspired by the structure of disulphide compounds which have advanced through various stages of clinical development, we designed a series of dithiodiglycolic acid derivatives. These were prepared from respective thiol synthons using an iodine- or benzotriazolyl chloride-promoted oxidative disulphide bond formation. Inhibition of TrxR present in cell lysates from human neuroblastoma cells (SH-SY5Y) and rat liver cells indicated several compounds with a potential for TrxR inhibition. Some of these compounds were also tested for growth inhibition against two human cancer cell lines and normal human keratinocytes.

## Graphical Abstract

**Figure:**
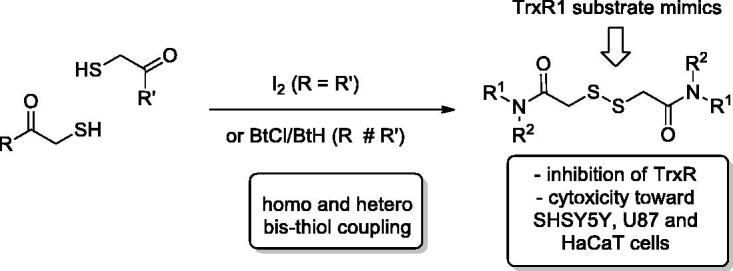

## Introduction

Thioredoxin reductase enzymes (TrxR, EC 1.8.1.9), which are the focus of this work, belong to thioredoxin system along with NADPH and thioredoxin (Trx). The system is highly conserved among species and responsible for regulating redox processes, gene transcription, and protection against reactive oxygen species (ROS)^1^. TrxR maintains Trx in its reduced bis-sulfhydryl state that, in turn, interacts with antioxidant enzymes and transcription factors^2^. TrxR1 is a cytosolic isoform of the enzyme (along with mitochondrial TrxR2 and TrxR3 expressed primarily in the testes). TrxR enzymes are overexpressed in cancer cells contributing to their resistant phenotype characterised by higher levels of ROS^3^. Targeting TrxR specifically with various, mostly electrophilic, inhibitors have thus become an attractive new approach for the development of anticancer therapy^4^.

**Figure 1.**
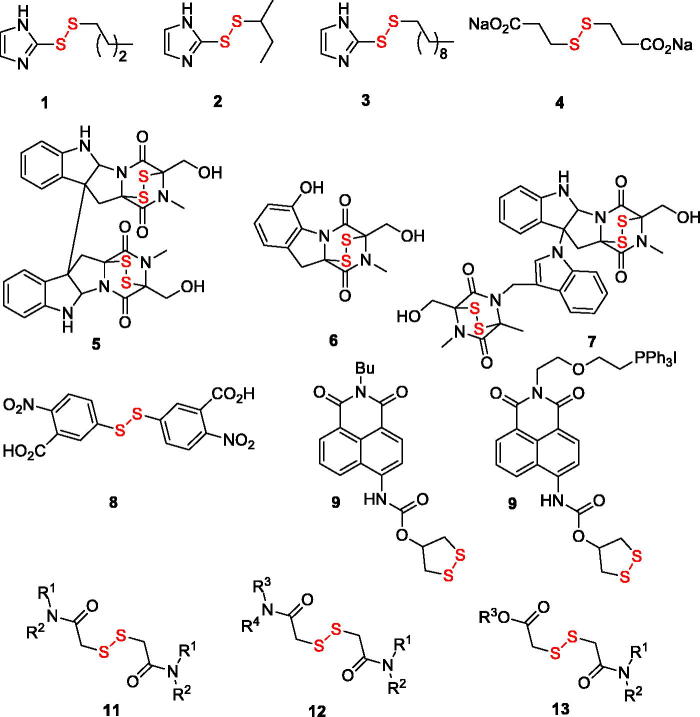
Known disulphide TrxR inhibitors (**1**–**7**) and fluorescent probes (**8**–**10**) as well as compounds explored in this work (**11**–**13**).

The various types of inhibitors targeting the catalytic selenocysteine residue of TrxR with varying degree of electrophilicity – Michael acceptors, disulphide and diselenide substrate mimics, metal-based inhibitors as well as compounds of miscellaneous structure – have been recently reviewed in comprehensive accounts by Bellelli^5^ and Fang^6^ ^,^ ^7^. Particularly relevant to the present study are the disulphide substrate analogues: e.g. alkyl 2-imidazolyl disulphides **1–3** ^8^ (**2**, whose *K*_i_ value toward the enzyme was determined as 31 µM, is also known as PX-12 and has been in Phase 1 clinical trials for advanced metastatic cancer and was stopped due to safety concerns^6^), disodium 2,2′-dithio-bis-ethane sulphonate **4** ^9^ (also known as Travocept^TM^ which advanced through phase III clinical trials in patients with non-small cell lung cancer and has a *K*_m_ value of 72 µM^6^) as well as bicyclic disulphide-containing natural products chaetocin (**5**), gliotoxin (**6**) and chaetomin (**7**) which have been shown to possess *K*_m_ values of 4.6 µM, 16.9 µM, and 16.1 µM, respectively – and have demonstrated a substrate-competitive inhibition profile toward TrxR1 and pro-apoptotic efficacy toward cancer cells^10^. Other disulphide compounds – 5,5′-dithiobis(2-nitrobenzoic acid) (**8** also known as DTNB or Ellman’s reagent)^11^, thioredoxin reductase fluorogenic substrate (**9** or TRFS-green)^12^ and mitochondria-targeted “mito-TRFS” (**10**)^13^ have not been employed as TrxR inhibitors but rather as fluorescent probes, also due to their being substrate (disulphide-linked Trx) analogues. Based on the structure of these disulphide TrxR ligands, we designed a series of symmetric (**11**) and non-symmetric (**12**–**13**) derivatives of dithiodiglycolic acid (Figure 1). Herein, we report the results of their synthetic exploration and biological testing for TrxR inhibition and cytotoxicity toward two human cancer cell lines and normal human keratinocytes.

## Materials and methods

### Chemical syntheses – general

NMR spectroscopic data were recorded with Bruker Avance 400 spectrometer (400.13 MHz for ^1^H and 100.61 MHz for ^13^C) and Bruker Avance 500 spectrometer (500.03 for ^1^H and 125 MHz for ^13^C) in DMSO-d_6_ and in CDCl_3_ and were referenced to residual solvent proton signals (*δ*_H_ = 2.50 and 7.26 ppm, respectively) and solvent carbon signals (*δ*_C_ = 39.5 and 77.0 ppm, respectively). Mass spectra were recorded with a Bruker Maxis HRMS-ESI-qTOF spectrometer (electrospray ionisation mode). Merck silica gel 60 mesh was used for column chromatography. TLC was performed with Macherey-Nagel Alugram Sil G/UV254 plates. Thioacetic acid, trityl chloride, methyl thioglycolate, amines, and 1*H*-benzotriazole, 2,2′-disulfanediyldiacetic acid (**14**) and 2,2′-disulfanediyldiacetic acid disodium salt (**15**) were obtained from commercial sources. 2-(Tritylthio)acetic acid (**16**) was prepared from thioacetic acid and trityl chloride according to literature procedure^14^. Dimethyl 2,2′-disulfanediyldiacetate (**17**) was prepared by oxidative dimerisation of methyl thioglycolate^15^. 1-Chlorobenzotriazole was prepared according to known procedure^16^. DCM was distilled from P_2_O_5_ and stored over MS 4 Å. THF was distilled from sodium benzophenone under Ar. All synthesised thiols and disulphides are air-sensitive, and therefore were stored in sealed vials at −20 °C. Characterisation data are provided only for one representative compound in each group. The rest of the compounds are described in the Supplementary Data.

## General procedure 1: preparation of 2-(tritylthio)acetamides 18a–l

To a solution of 2-(tritylthio)acetic acid **16** (1.5–4.5 mmol) in dry dichloromethane (DCM; 5 ml/mmol) 1,1′-carbonyldiimidazole (CDI; 1.0–1.1 equiv.) was added portionwise (gas evolution!). After stirring for 50 min, amine (1.0–1.1 equiv.) was added and the reaction mixture was left stirring at r.t. overnight. The reaction mixture was washed with citric acid (2 N aq. solution), saturated aq. NaHCO_3_ and water. Organic layer was dried (over Na_2_SO_4_), filtered, and concentrated *in vacuo* to give pure title amides.

**1-(Pyrrolidin-1-yl)-2-(tritylthio)ethan-1-one (18a)** was prepared according to General Procedure 1 from 2-(tritylthio)acetic acid (668 mg, 2 mmol), pyrrolidine (150 mg, 2.1 mmol) and CDI (340 mg, 2.1 mmol). Yield 584 mg, 75%. White solid. ^1^H NMR (400 MHz, CDCl_3_) δ 7.57–7.43 (m, 6H), 7.37–7.28 (m, 6H), 7.28–7.17 (m, 3H), 3.40 (t, *J* = 6.6 Hz, 2H), 3.07 (t, *J* = 6.4 Hz, 2H), 2.93 (s, 2H), 1.85–1.77 (m, 4H).^13^C NMR (101 MHz, CDCl_3_) δ 166.8, 144.3, 129.6, 128.0, 126.8, 66.8, 46.5, 46.0, 36.1, 26.0, 24.3. HRMS (ESI), *m/z* calcd for C_25_H_25_NOSNa [M + Na]^+^ 410.1549, found 410.1553.

## General procedure 2: preparation of thiols 19a–c

To a stirred solution of protected amide **18a–c** (2 mmol) in TFA (5 ml) triethylsilane (TES; 1.1 equiv.) was added at room temperature. Reaction progress was monitored by TLC and reaction mixture turned colourless from deep yellow. After 5–30 min, reaction mixture was washed, diluted with water (15 ml), and extracted with hexane (3 × 15 ml). The aqueous layer was then extracted with DCM (3 × 15 ml). The combined organic layer was dried over Na_2_SO_4_, filtered and concentrated to provide pure title compounds^17^.

**2-Mercapto-1-(pyrrolidin-1-yl)ethan-1-one (19a)** was prepared according to General Procedure 2 from **18a** (774 mg, 2 mmol) and TES (254 mg, 2.2 mmol). Yield 178 mg, 61%. ^1^H NMR (400 MHz, CDCl_3_) δ 3.57 (q, *J* = 7.3 Hz, 4H), 3.32 (s, 2H), 2.23 (s, 1H), 2.07 (p, *J* = 6.8 Hz, 2H), 1.96 (p, *J* = 6.8 Hz, 2H). 13C NMR (101 MHz, CDCl_3_) δ 170.7, 47.4, 47.0, 26.3, 25.9, 24.3. HRMS (ESI), *m/z* calcd for C_6_H_12_NOS [M + H]^+^ 146.0634, found 146.0634.

## General procedure 3: preparation of thiols 19d–k

To a stirred solution of protected amide **18d–k** (1.2–2.1 mmol) in DCM (10 ml) trifluoroacetic acid (TFA; 5 equiv.) was added followed by triethylsilane (TES; 2 equiv.). Reaction progress was monitored by TLC. After 2–16 h, reaction mixture was washed with water (3 × 30 ml), dried and concentrated *in vacuo.* The residue was suspended in hexane (10 ml) and sonicated using ultrasonic bath, cooled and filtered to give corresponding thiol^18^.

***N-*Cyclopropyl-2-mercaptoacetamide (19d)** was prepared according to General Procedure 3 from **18d** (746 mg, 2 mmol), TFA (1.27 g, 5.9 mmol) and TES (514 mg, 4.4 mmol). Yield 233 mg, 89% (85% NMR purity). ^1^H NMR (400 MHz, CDCl_3_) δ 6.72 (s, 1H), 3.23 (d, *J* = 9.1 Hz, 2H), 2.76 (dq, *J* = 7.1, 3.5 Hz, 1H), 1.87 (t, *J* = 9.1 Hz, 1H), 0.88–0.79 (m, 2H), 0.60–0.52 (m, 2H). HRMS (ESI), *m/z* calcd for C_5_H_9_NOSNa [M + Na]^+^ 154.0297, found 154.0299.

## General procedure 4: preparation of symmetric disulphides 11a–k

To the solution of thiol **19a–k** (0.3–0.9 mmol) in DCM and triethylamine (TEA; 1.5–3 equiv.) solid I_2_ was added portionwise until persistent deep yellow colour appeared. After stirring for 5 min, the conversion of thiol was checked by TLC. In case of compounds **11a–d, f, g, k** the reaction mixture was concentrated and the residue was crystallised from hexane-ethyl acetate to give pure disulphides. Compounds **11e, h, i, j** precipitated from the reaction mixture and were isolated by filtration and washing with DCM (2 × 5 ml)^19^.

**2,2′-Disulfanediylbis(1-(pyrrolidin-1-yl)ethan-1-one) (11a)** was prepared according to General Procedure 4 from **19a** (90 mg, 0.31 mmol) and TEA (66 mg, 0.93 mmol). Yield 66 mg, 75%. ^1^H NMR (400 MHz, CDCl_3_) δ 3.69 (s, 4H), 3.57 (t, *J* = 6.8 Hz, 4H), 3.51 (t, *J* = 6.9 Hz, 4H), 2.04–1.96 (m, 4H), 1.95–1.84 (m, 4H). 13C NMR (126 MHz, CDCl_3_) δ 166.7, 47.2, 46.2, 42.3, 26.2, 24.4. HRMS (ESI), *m/z* calcd for C_12_H_20_N_2_O_2_S_2_Na [M + Na]^+^ 311.0858, found 311.0873.

## General procedure 5: preparation of non-symmetric disulphides 12(13)a

A mixture of 1-chlorobenzotriazole (BtCl; 1.1 equiv.) and 1*H*-benzotriazole (BtH; 1 equiv.) was placed into a Schlenk tube, which was then evacuated and filled with argon three times followed by addition of dry DCM (10 ml) and cooling to −78 °C. First thiol (0.5–0.6 mmol) dissolved in 5 ml DCM was added dropwise during 15–20 min to form a yellow solution. Then the reaction mixture was slowly warmed to −20 °C and the second thiol (1.1 equiv.) dissolved in 5 ml DCM was added dropwise during 15–20 min. The resulting solution was left stirring at 0 °C for 30 min. The reaction mixture was then quenched with aqueous Na_2_S_2_O_3_, washed with saturated aq. NaHCO_3_ (20 ml) and extracted with DCM (3 × 50 ml). Combines organic layer was dried over Na_2_SO_4_, filtered and concentrated to give crude material, which was purified by silica gel column chromatography using DCM/MeOH mixture (100:1) as eluent^20^.

**2-((2-Morpholino-2-oxoethyl)disulfanyl)-*N*-propylacetamide (12a)** was prepared according to General Procedure 5 from **19d** (84 mg, 0.63 mmol) as the first thiol, **19 b** (102 mg, 0.63 mmol) as the second thiol, BtCl (106 mg, 0.69 mmol) and BtH (75 mg, 0.63 mmol). Yield 70 mg, 38%. ^1^H NMR (400 MHz, CDCl_3_) δ 3.73 (dt, *J* = 6.4, 3.8 Hz, 4H), 3.69 (d, *J* = 6.2 Hz, 2H), 3.67 (s, 2H), 3.53 (t, *J* = 4.9 Hz, 2H), 3.50 (s, 2H), 3.35–3.26 (m, 2H), 1.62 (h, *J* = 7.4 Hz, 2H), 0.98 (t, *J* = 7.4 Hz, 3H). 13C NMR (126 MHz, CDCl_3_) δ 167.8, 167.0, 66.8, 66.6, 46.8, 42.9, 42.5, 41.8, 39.9, 22.8, 11.5. HRMS (ESI), *m/z* calcd for C_11_H_20_N_2_O_3_S_2_Na [M + Na]^+^ 315.0808, found 315.0815.

**Methyl 2-((2-((4-fluorophenyl)amino)-2-oxoethyl)disulfanyl)acetate (13a)** was prepared according to General Procedure 5 from **19i** (110 mg, 0.59 mmol) as the first thiol, methyl thioglycoate (69 mg, 0.65 mmol) as the second thiol, BtCl (100 mg, 0.65 mmol) and BtH (71 mg, 0.6 mmol). Yield 68 mg, 40%. ^1^H NMR (400 MHz, CDCl_3_) δ 8.71 (s, 1H), 7.67–7.43 (m, 2H), 7.05 (m, 2H), 3.84 (s, 3H), 3.65 (s, 2H), 3.60 (s, 2H). 13C NMR (101 MHz, CDCl_3_) δ 171.31, 166.03, 159.51 (d, *J_CF_* = 243.7 Hz), 133.79 (d, *J_CF_* = 2.9 Hz), 121.69 (d, *J_CF_* = 7.8 Hz), 115.63 (d, *J_CF_* = 22.5 Hz), 53.17, 42.85, 41.87. HRMS (ESI), *m/z* calcd for C_11_H_12_FNO_3_S_2_Na [M + Na]^+^ 312.0135, found 312.0139.

## Cell culture

Human neuroblastoma cells (SH-SY5Y) were grown in 1:1 mixture of Eagle's Minimum Essential Medium and F12 Medium (Sigma) supplemented with 10% foetal bovine serum (Sigma). Human keratinocytes (HaCaT) were grown in DMEM, supplemented with 10% foetal bovine serum, 2 mM L-glutamine, 5000 U/ml penicillin, and 5 mg/ml streptomycin. Human glioblastoma cell (U87-MG) were grown in Minimum Essential Media (MEM), containing 10% foetal bovine serum, 2 mM L-glutamine, 5,000 U/ml penicillin, and 5 mg/ml streptomycin. All cell lines were obtained from American Type Culture Collection (ATCC, Rockville, MD). All cell lines were grown at 37 °C in a humidified 5% CO_2_ atmosphere.

## TrxR inhibition assay

For TrxR assay, lysate from SH-SY5Y cells and rat liver were used. SHSY5Y cells were washed twice with phosphate buffer solution (PBS) and harvested from the flask with a scraper. Cells were homogenised in ice-cold 50 mM potassium phosphate buffer (pH 7.4), containing 1 mM EDTA, with Ultra Turrax T25 (Ikawerk, Janke and Kunkel Inc., Staufen, Germany). The homogenates were centrifuged at 10,000 × g for 15 min at 4 °C. The protein concentrations of the supernatants were determined using a Bio-Rad protein assay kit (Bio-Rad Laboratories, USA). TrxR activity was assayed using 5,5-dithio-bis-(2-nitrobenzoic acid) (DTNB, Sigma-Aldrich, USA) as substrate^21^. The reactions were run on a 96-well plates, final volume of 100 µl in 50 mM potassium phosphate buffer (pH 7.0), containing 50 µg of the cell lysate proteins, 1-mM EDTA, 50 mM KCl, 0.2 mg/ml bovine serum albumin, 0.25 mM NADPH (Acros Organics, USA). Reaction mixture was incubated for 15 min at room temperature in plate shaker (PST-60HL-4, BioSan, Riga, Latvia); afterwards DTNB was added to the total concentration of 2.5 mM. Enzyme kinetics was monitored on a Tecan Infinite1000 microplate reader, by measuring the increase in absorbance at 412 nm for 20 min. The background TrxR-independent reduction of DTNB in the cell lysates, as determined in the presence of aurothiomalate (200 µM), was subtracted from each value^22^. Reaction mixture with rat liver lysate contained 35 µg of proteins, 1 mM EDTA, 50 mM KCl, 0.2 mg/ml bovine serum albumin, 0.25 mM NADPH, and 2.5 mM DTNB. Reactions with rat liver lysate were run in the same manner as cell lysates.

## Cytotoxicity assay

Cells grown in 25 cm^2^ tissue flasks were trypsinized, seeded into flat-bottomed adherent 96-well cell culture plates in appropriate medium (SH-SY5Y 12,000 cells/well, U87-MG 4000 cells/well, HaCaT 2000 cells/well) and incubated overnight; afterwards the cells were exposed to different concentrations of the tested compounds for 72 h. Cell viability was measured by MTT assay^23^. Briefly, after incubation with the compounds, medium containing 0.2 mg/ml MTT was added. After incubation (3 h, 37 °C, 5% CO_2_), the MTT-containing medium was removed, and 200 µl of dimethyl sulfoxide (DMSO) was immediately added to each sample. The samples were assessed at 540 nm on LKB 5060–006 Microplate Reader (Vienna, Austria) microplate reader. The half-maximal inhibitory concentration (IC_50_) of each compound was calculated using Graph Pad Prism® 6.0 (GraphPad Software, Inc., USA).

## Results and discussion

### Chemistry

**Scheme 1.**
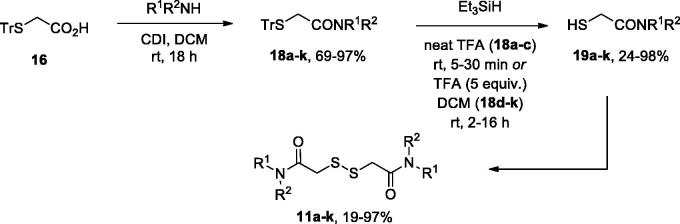
Preparation of symmetric disulphides **11a–k**.

Initial attempts to elaborate commercially available dithiodiglycolic acid (**14**) into compounds **11**–**13** failed, since either HATU- or DCC-mediated amidation of it resulted in a difficult-to separate mixture of mono- and symmetric bis-amides of **14** (also containing **14** itself). Therefore, for the preparation of symmetric bis-amides **11a–k** we adopted the route depicted in Scheme 1. Trityl-protected thioacetic acid (**16**)^14^ was activated by 1,1′-carbonyldiimidazole (CDI) to give amides **18a–k** in good to excellent yields. The trityl group was removed by the action of trifluoroacetic acid (TFA) used as a solvent (**18a–c**)^18^ or as a reagent (5 equiv.) in DCM (**18d–k**)^19^, in the presence of triethylsilane. The reaction was completed in 5–30 min in the former case and took 2–16 h – in the latter. The free thiols **19a–k** thus obtained (in moderate to excellent yield) were homo-coupled on the action of molecular iodine in the presence of triethylamine^19^ to give target compounds **11a–k** in moderate to excellent yield.

**Scheme 2.**
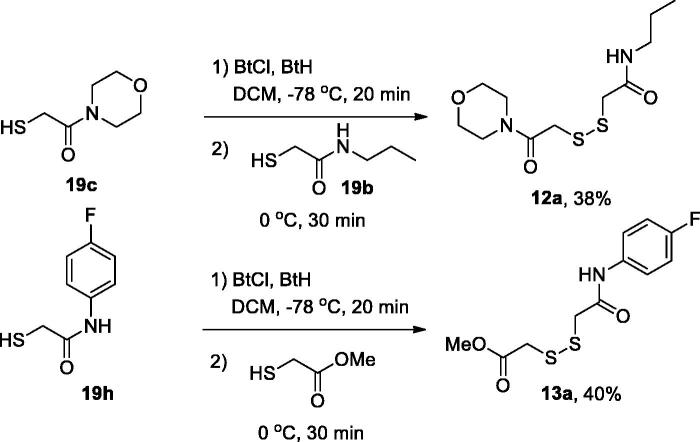
Preparation of non-symmetric disulphides **12(13)a**.

For the preparation of non-symmetric disulphides **12(13)a**, one of the thiols **19** was activated at −78 °C by the action of a roughly equimolar mixture of *N*-chlorobenzotriazole (BtCl) and benzotriazole (BtH), and the resulting intermediate was treated with a second thiol **19** (**12a**) or methyl mercaptoacetate (**13a**) to give, on stirring at 0 °C for 30 min, respective disulphides (Scheme 2).

Biological evaluation of compounds **11a–k**, **12a**, and **13a** as well as commercially available dithiodiglycolic acid (**14**), its disodium salt (**15**) and dimethyl ester (**17**) against human neuroblastoma (SHSY5Y) as well as rat liver cell lysates using DTNB assay^21^ revealed that a number of compounds (**11a–d**, **11 g**, **11j–k**, **13a**, **14**, and **17**) possessed double-digit micromolar activity against liver cell lysate while their inhibitory activity measured against neuroblastoma (SHSY5Y) cell lysate was significantly lower (compounds **11a–b**, **11 g**, **11j**, **14**, and **17** displaying virtually no activity). Considering that TrxR1 expression levels in hepatocytes are significantly higher compared to SHSY5Y cells^24^ ^,^ ^25^, these results may suggest higher activity of our novel compounds towards lysates with higher TrxR1 content. Notably, compound **15** which is a close analogue (double homolog) of compound **4** (Travocept^TM^ which entered Phase III clinical trials in 2015^6^) is completely devoid of inhibitory activity toward TrxR.

Selected compounds from the active cohort (**11 b**, **11d**, **11g**) and also compound **12a** (which displayed identical inhibitory potency with IC_50_ around 45 µM against SHSY5Y and hepatocyte lysates) were tested for cytotoxicity against neuroblastoma (SHSY5Y), glioblastoma (U87), and immortal keratinocyte (HaCaT) cells. Interestingly, compound **11 b** was found to be 2–3 times more active against U87 cell line compared to the other three compounds (Table 1).

**Table 1.** Inhibition profile of compounds prepared in this work against TrxR1 measured in neuroblastoma (SHSY5Y) and hepatocyte lysates; cytotoxicity profile of selected compounds determined against SHSY5Y, U87, and HaCaT cells.

Compound	Structure	TrxR IC_50_ (μM)^a^		CC_50_ (μM)^a^		
		SHSY5Y lysate	Hepatocyte lysate	SHSY5Y	U87	HaCaT
**11a**		>200	170.4 ± 54.2	nd	nd	nd
**11b**		>200	26.5 ± 2.9	219.7 ± 15.3	54.6 ± 3.4	58.4 ± 5.0
**11c**		189.5 ± 78.4	182.5 ± 32.2	nd	nd	nd
**11d**		63.6 ± 27.5	34.7 ± 9.1	202.3 ± 12.0	179.6 ± 12.6	69.0 ± 3.4
**11e**		>200	>200	nd	nd	nd
**11f**		>200	>200	nd	nd	nd
**11g**		>200	22.2 ± 3.1	174 ± 18.3	125.2 ± 15.6	43.0 ± 2.9
**11h**		>200	>200	nd	nd	nd
**11i**		>200	>200	nd	nd	nd
**11j**		>200	90.4 ± 18.7	nd	nd	nd
**11k**		>200	24.0 ± 11.5	nd	nd	nd
**12a**		44.4 ± 15.1	45.6 ± 6.8	176.3 ± 13.4	186.8 ± 15.9	71.8 ± 6.9
**13a**		186.4 ± 83.8	>200	nd	nd	nd
**14**		>200	>200	nd	nd	nd
**15**		>200	>200	nd	nd	nd
**17**		>200	>200	nd	nd	nd

^a^The values provided are mean ± SEM (*n* = 3).“nd”: not determined; IC: inhibitory concentration; CC: cytotoxicity concentration.

## Conclusions

We have described design, synthesis, and biological evaluation of a series of dithiodiglycolic acid derivatives. Symmetric bis-amides were prepared via molecular iodine-promoted bis-homocoupling of thioglycolic acid amides, while non-symmetric diamide and amide-ester were prepared via bis-thiol heterocoupling promoted by *N*-chorobenzotriazole/benzotriazole mixture. The compound set tested against human neuroblastoma (SHSY5Y) and rat liver cell lysates for TrxR inhibition using DTNB assay revealed a promising inhibitory profile for 6 out 16 compounds tested, namely, higher inhibitory potency toward hepatocyte lysate TrxR1 compared to SHSY5Y lysate. Several of active compounds also showed cytotoxic effect toward human neuroblastoma (SHSY5Y) and glioblastoma (U87), as well as, immortal human keratinocyte (HaCaT) cell lines. Thus, the derivatives studied in this work represent a novel lead chemotype that could be further developed into more potent and more specific TrxR1 inhibitors valuable for anticancer therapy.

## Supplementary Material

Supplemental Material
