# Supplementary material for: Design, synthesis, and biological evaluation of novel derivatives of dithiodiglycolic acid prepared via oxidative coupling of thiols
Source: J Enzyme Inhib Med Chem. 2019 Feb 12;34(1):665–71. doi: 10.1080/14756366.2019.1575372 (PMC6374954; doi:10.1080/14756366.2019.1575372)
Supplement: Supplemental Material [file IENZ_A_1575372_SM5470.docx]

Supporting Information

for

**Novel derivatives of dithiodiglycolic acid prepared via oxidative coupling of thiols inhibit thioredoxin reductase and cancer cell growth**

Olga Bakulina, Anton Bannykh, Mirna Jovanović, Ilona Domračeva, Ana Podolski-Renić, Raivis Žalubovskis, Milica Pešić, Dmitry Dar’in and Mikhail Krasavin*^*^*

Contents

| Characterization data for compounds **18a-k, 19a-k, 11a-k, 12a** and **13a** | S2-S10 |
| --- | --- |
| Copies of ^1^H and ^13^C NMR spectra | S11-S41 |

**1-(Pyrrolidin-1-yl)-2-(tritylthio)ethan-1-one (18a)** was prepared according to General Procedure 1 from 2-(tritylthio)acetic acid (668 mg, 2 mmol), pyrrolidine (150 mg, 2.1 mmol) and CDI (340 mg, 2.1 mmol). Yield 584 mg, 75 %. White solid.^1^H NMR (400 MHz, CDCl_3_) δ 7.57 – 7.43 (m, 6H), 7.37 – 7.28 (m, 6H), 7.28 – 7.17 (m, 3H), 3.40 (t, *J* = 6.6 Hz, 2H), 3.07 (t, *J* = 6.4 Hz, 2H), 2.93 (s, 2H), 1.85 – 1.77 (m, 4H).^13^C NMR (101 MHz, CDCl_3_) δ 166.8, 144.3, 129.6, 128.0, 126.8, 66.8, 46.5, 46.0, 36.1, 26.0, 24.3. HRMS (ESI), m/z calcd for C_25_H_25_NOSNa [M+Na]^+^ 410.1549, found 410.1553.

***N*-Propyl-2-(tritylthio)acetamide (18b)** was prepared according to General Procedure 1 from 2-(tritylthio)acetic acid (1.34 g, 4 mmol), *n*-propyl amine (236 mg, 4 mmol) and CDI (648 mg, 4 mmol). Yield 1.3 g, 90 %. White solid. ^1^H NMR (400 MHz, CDCl_3_) δ 7.51 – 7.40 (m, 6H), 7.36 – 7.30 (m, 6H), 7.29 – 7.19 (m, 3H), 6.04 (s, 1H), 3.15 (s, 2H), 3.01 – 2.87 (m, 2H), 1.38 (q, *J* = 7.3 Hz, 2H), 0.85 (t, *J* = 7.4 Hz, 3H). ^13^C NMR (101 MHz, CDCl_3_) δ 167.8, 144.1, 129.5, 128.2, 127.1, 67.9, 41.4, 36.0, 22.4, 11.3. HRMS (ESI), m/z calcd for C_24_H_25_NOS [M+Na]^+^ 398.1549, found 398.1546.

**1-Morpholino-2-(tritylthio)ethan-1-one (18c)** was prepared according to General Procedure 1 from 2-(tritylthio)acetic acid (1.34 g, 4 mmol), morpholine (365 mg, 4.2 mmol) and CDI (648 mg, 4 mmol). Yield 1.53 g, 95 %. White solid.^1^H NMR (400 MHz, CDCl_3_) δ 7.54 – 7.43 (m, 6H), 7.33 (m, 6H), 7.31 – 7.17 (m, 3H), 3.62 (q, *J* = 4.0, 3.3 Hz, 2H), 3.54 (dt, *J* = 9.4, 4.8 Hz, 4H), 3.04 (t, *J* = 4.8 Hz, 2H), 2.95 (s, 2H). ^13^C NMR (101 MHz, CDCl_3_) δ 167.1, 144.0, 129.5, 128.1, 126.9, 67.2, 66.7, 66.7, 46.4, 42.2, 34.4. HRMS (ESI), m/z calcd for C_25_H_25_NO_2_SNa [M+Na]^+^ 426.1498, found 426.1503.

***N*-Cyclopropyl-2-(tritylthio)acetamide (18d)** was prepared according to General Procedure from 2-(tritylthio)acetic acid (900 mg, 2.7 mmol), cyclopropyl amine (169 mg, 3 mmol) and CDI (476 mg, 2.9 mmol). Yield 873 mg, 88 %. White solid. ^1^H NMR (400 MHz, CDCl_3_) δ 7.43 (m, 6H), 7.36 – 7.30 (m, 6H), 7.29 – 7.22 (m, 3H), 6.07 (s, 1H), 3.13 (s, 2H), 2.41 (tq, *J* = 7.2, 3.6 Hz, 1H), 0.65 (td, *J* = 7.0, 5.2 Hz, 2H), 0.36 – 0.19 (m, 2H). ^13^C NMR (101 MHz, CDCl_3_) δ 169.4, 144.0, 129.4, 128.2, 127.1, 67.9, 35.7, 22.6, 6.2. HRMS (ESI), m/z calcd for C_24_H_23_NOSNa [M+Na]^+^ 396.1393, found 396.1400.

***N*-(4-Methoxybenzyl)-2-(tritylthio)acetamide (18e)** was prepared according to General Procedure 1 from 2-(tritylthio)acetic acid (500 mg, 1.5 mmol), 4-methoxybenzyl amine (221 mg, 1.65 mmol) and CDI (267 mg, 1.65 mmol). Yield 554 mg, 81 %. White solid. ^1^H NMR (400 MHz, CDCl_3_) δ 7.71 (s, 1H), 7.53 – 7.43 (m, 6H), 7.38 – 7.28 (m, 6H), 7.26 – 7.16 (m, 5H), 6.87 – 6.76 (m, 2H), 3.80 (s, 3H), 3.31 (s, 2H). 13C NMR (101 MHz, CDCl3) δ 167.7, 159.1, 144.0, 129.7, 129.5, 129.2, 128.2, 127.1, 114.0, 68.0, 55.3, 43.3, 36.0. HRMS (ESI), m/z calcd for C_29_H_27_NO_2_SNa [M+Na]^+^ 476.1655, found 476.1671.

**1-(4-Methylpiperazin-1-yl)-2-(tritylthio)ethan-1-one (18f)** was prepared according to General Procedure from 2-(tritylthio)acetic acid (700 mg, 2.1 mmol) and methylpiperazine (231 mg, 2.3 mmol) and CDI (360 mg, 2.2 mmol). Yield 758 mg, 87 %. White solid. ^1^H NMR (500 MHz, CDCl_3_) δ 7.54 – 7.47 (m, 6H), 7.32 (m, 6H), 7.28 – 7.20 (m, 3H), 3.62 – 3.54 (m, 2H), 3.11 – 3.03 (m, 2H), 2.94 (s, 2H), 2.34 (t, *J* = 5.2 Hz, 2H), 2.27 (s, 3H), 2.26 (d, *J* = 5.1 Hz, 2H). ^13^C NMR (126 MHz, CDCl_3_) δ 166.8, 144.0, 129.5, 128.1, 126.9, 67.1, 55.2, 54.5, 45.9, 41.7, 34.6. HRMS (ESI), m/z calcd for C_26_H_28_N_2_OSNa [M+Na]^+^ 439.1815, found 439.1827.

***N*-(*tert-*Butyl)-2-(tritylthio)acetamide (18g)** was prepared according to General Procedure 1 from 2-(tritylthio)acetic acid (900 mg, 2.7 mmol), *tert*-butyl amine (214 mg, 2.9 mmol) and CDI (476 mg, 2.9 mmol). Yield 720 mg, 69 %. White solid. ^1^H NMR (400 MHz, CDCl_3_) δ 7.49 – 7.40 (m, 6H), 7.36 – 7.30 (m, 6H), 7.29 – 7.23 (m, 3H), 5.94 (s, 1H), 3.06 (s, 2H), 1.21 (s, 9H). ^13^C NMR (101 MHz, CDCl_3_) δ 167.0, 144.2, 129.5, 128.2, 127.0, 67.9, 51.2, 36.9, 28.4. HRMS (ESI), m/z calcd for C_25_H_27_NOSNa [M+Na]^+^ 412.1706, found 412.1716.

***N*-(4-Fluorophenyl)-2-(tritylthio)acetamide (18h)** was prepared according to General Procedure 1 from 2-(tritylthio)acetic acid (500 mg, 1.5 mmol), 4-fluoroaniline (183 mg, 1.65 mmol) and CDI (267 mg, 1.65 mmol). Yield 535 mg, 83 %. White solid. ^1^H NMR (400 MHz, CDCl_3_) δ 7.8 (s, 1H), 7.5 – 7.4 (m, 6H), 7.3 (m, 6H), 7.2 (dd, *J =* 6.0, 4.1 Hz, 5H), 7.0 (t, *J =* 8.7 Hz, 2H), 3.3 (s, 2H). ^13^C NMR (101 MHz, CDCl3) δ 165.9, 159.4 (d, *J_CF_* = 243.5 Hz), 143.8, 133.4, 129.4, 128.3, 127.2, 121.4 (d, *J_CF_* = 7.9 Hz), 115.4 (d, *J_CF_* = 22.5 Hz), 68.2, 36.5. HRMS (ESI), m/z calcd for C_27_H_22_FNOSNa [M+Na]^+^ 450.1298, found 450.1312.

***N*-(4-Methoxyphenyl)-2-(tritylthio)acetamide (18i)** was prepared according to General Procedure 1 from 2-(tritylthio)acetic acid (500 mg, 1.5 mmol), 4-methoxyaniline (203 mg, 1.65 mmol) and CDI (267 mg, 1.65 mmol). Yield 570 mg, 87 %. Pale purple solid. ^1^H NMR (400 MHz, CDCl_3_) δ 7.71 (s, 1H), 7.55 – 7.44 (m, 6H), 7.37 – 7.26 (m, 6H), 7.25 – 7.12 (m, 5H), 6.87 – 6.77 (m, 2H), 3.80 (s, 3H), 3.31 (s, 2H). ^13^C NMR (101 MHz, CDCl_3_) δ 165.8, 156.4, 143.9, 130.5, 129.4, 128.3, 127.2, 121.5, 113.9, 68.2, 55.4, 36.5. HRMS (ESI), m/z calcd for C_26_H_28_N_2_OSNa [M+Na]^+^ 439.1815, found 439.1832.

**2-(Tritylthio)acetamide (18j)**. 2-(Tritylthio)acetic acid (1 g, 3 mmol) was dissolved in dry THF followed by portionwise addition of CDI (534 mg, 3.3 mmol). The resulting solution was saturated with dry ammonia gas and was left stirring overnight. The reaction mixture was concentrated in vacuo and the residue was partitioned between DCM and 2N citric acid. The organic layer was washed with water, dried over Na_2_SO_4_ and concentrated in vacuo to give pure title compound. Yield 753 mg, 75 %. White solid. ^1^H NMR (400 MHz, CDCl_3_) δ 7.51 – 7.41 (m, 6H), 7.33 (m, 6H), 7.30 – 7.16 (m, 3H), 5.85 (s, 1H), 5.17 (s, 1H), 3.12 (s, 2H). ^13^C NMR (101 MHz, CDCl_3_) δ 171.0, 144.0, 129.5, 128.2, 127.1, 67.9, 35.6. HRMS (ESI), m/z calcd for C_21_H_19_NOSNa [M+Na]^+^ 356.1080, found 356.1093.

***N*-(Furan-2-ylmethyl)-2-(tritylthio)acetamide (18k)** was prepared according to General Procedure from 2-(tritylthio)acetic acid (1.5 g, 4.5 mmol) and furfurylamine (0.480 g, 4.9 mmol). Yield 1.8 g, 97 %. Beige solid. ^1^H NMR (400 MHz, CDCl_3_) δ 7.46 – 7.41 (m, 6H), 7.36 (d, *J* = 1.9 Hz, 1H), 7.33 – 7.27 (m, 6H), 7.27 – 7.20 (m, 3H), 6.33 (dd, *J* = 3.2, 1.9 Hz, 1H), 6.26 (s, 1H), 6.15 (d, *J* = 3.2 Hz, 1H), 4.14 (d, *J* = 5.5 Hz, 2H), 3.18 (s, 2H). ^13^C NMR (101 MHz, CDCl_3_) δ 167.7, 150.7, 144.0, 142.2, 129.5, 128.2, 127.1, 110.4, 107.6, 68.0, 36.7, 35.8. HRMS (ESI), m/z calcd for C_26_H_23_NO_2_SNa [M+Na]^+^ 436.1342, found 436.1356.

**2-Mercapto-1-(pyrrolidin-1-yl)ethan-1-one (19a)** was prepared according to General Procedure 2 from **18a** (774 mg, 2 mmol) and TES (254 mg, 2.2 mmol). Yield 178 mg, 61 %. ^1^H NMR (400 MHz, CDCl_3_) δ 3.57 (q, *J* = 7.3 Hz, 4H), 3.32 (s, 2H), 2.23 (s, 1H), 2.07 (p, *J* = 6.8 Hz, 2H), 1.96 (p, *J* = 6.8 Hz, 2H). ^13^C NMR (101 MHz, CDCl_3_) δ 170.7, 47.4, 47.0, 26.3, 25.9, 24.3. HRMS (ESI), m/z calcd for C_6_H_12_NOS [M+H]^+^ 146.0634, found 146.0634.

**2-Mercapto-*N*-propylacetamide (19b)** was prepared according to General Procedure 2 from **18b** (726 mg, 2 mmol) and TES (254 mg, 2.2 mmol). Yield 168 mg, 63 %. ^1^H NMR (400 MHz, CDCl_3_) δ 6.70 (s, 1H), 3.37 – 3.21 (m, 4H), 1.87 (t, *J* = 9.1 Hz, 1H), 1.64 – 1.54 (m, 2H), 0.97 (t, *J* = 7.4 Hz, 3H). ^13^C NMR (101 MHz, CDCl_3_) δ 169.0, 41.6, 28.3, 22.7, 11.3. HRMS (ESI), m/z calcd for C_5_H_11_NOSNa [M+Na]^+^ 156.0454, found 156.0457.

**2-Mercapto-1-morpholinoethan-1-one (19c)** was prepared according to General Procedure 2 from **18c** (806 mg, 2 mmol) and TES (254 mg, 2.2 mmol). Yield 251 mg, 78 %. 1H NMR (400 MHz, CDCl3) δ 3.73 (dt, *J* = 11.2, 5.0 Hz, 4H), 3.66 (t, *J* = 4.9 Hz, 2H), 3.51 (t, *J* = 4.8 Hz, 2H), 3.36 (d, *J* = 6.9 Hz, 2H), 2.12 (t, *J* = 7.7 Hz, 1H). ^13^C NMR (101 MHz, CDCl_3_) δ 168.7, 66.7, 66.5, 46.7, 42.5, 25.8. HRMS (ESI), m/z calcd for C_6_H_11_NO_2_SNa [M+Na]^+^ 184.0403, found 184.0409.

***N-*Cyclopropyl-2-mercaptoacetamide (19d)** was prepared according to General Procedure 3 from **18d** (746 mg, 2 mmol), TFA (1.27 g, 5.9 mmol) and TES (514 mg, 4.4 mmol). Yield 233 mg, 89 % (85% NMR purity). ^1^H NMR (400 MHz, CDCl_3_) δ 6.72 (s, 1H), 3.23 (d, *J* = 9.1 Hz, 2H), 2.76 (dq, *J* = 7.1, 3.5 Hz, 1H), 1.87 (t, *J* = 9.1 Hz, 1H), 0.88 – 0.79 (m, 2H), 0.60 – 0.52 (m, 2H). HRMS (ESI), m/z calcd for C_5_H_9_NOSNa [M+Na]^+^ 154.0297, found 154.0299.

**2-Mercapto-*N*-(4-methoxybenzyl)acetamide (19e)** was prepared according to General Procedure 3 from **18e** (530 mg, 1.2 mmol), TFA (670 mg, 11 mmol) and TES (267 mg, 2.3 mmol). Yield 216 mg, 87 %. ^1^H NMR (400 MHz, CDCl_3_) δ 7.27 – 7.22 (m, 2H), 6.94 – 6.87 (m, 2H), 4.43 (d, *J* = 5.6 Hz, 2H), 3.83 (s, 3H), 3.30 (d, *J* = 9.1 Hz, 2H), 1.88 (t, *J* = 9.1 Hz, 1H). ^13^C NMR (101 MHz, CDCl_3_) δ 168.9, 159.2, 129.8, 129.2, 114.2, 55.3, 43.5, 28.3. HRMS (ESI), m/z calcd for C_10_H_13_NO_2_SNa [M+Na]^+^ 234.0559, found 234.0565.

**2-Mercapto-1-(4-methylpiperazin-1-yl)ethan-1-one (19f)** was prepared according to General Procedure 3 from **18f** (750 mg, 1.8 mmol), TFA (1 g, 9 mmol) and TES (417 mg, 3.6 mmol). Yield 75 mg, 24 % (80% NMR purity). ^1^H NMR (400 MHz, CDCl_3_) δ 3.67 (t, *J* = 5.2 Hz, 2H), 3.52 (t, *J* = 5.1 Hz, 2H), 3.36 (d, *J* = 7.5 Hz, 2H), 2.47 (t, *J* = 5.1 Hz, 2H), 2.42 (t, *J* = 5.2 Hz, 2H), 2.34 (s, 3H), 2.11 (t, *J* = 7.6 Hz, 1H). HRMS (ESI), m/z calcd for C_7_H_14_N_2_OSNa [M+Na]^+^ 175.0900, found 175.0905.

***N*-(*tert*-Butyl)-2-mercaptoacetamide (19g)** was prepared according to General Procedure 3 from **18g** (700 mg, 1.8 mmol), TFA (1 g, 9 mmol) and TES (417 mg, 3.6 mmol). Yield 140 mg, 53 % (80% NMR purity). ^1^H NMR (400 MHz, CDCl_3_) δ 6.47 (s, 1H), 3.17 (d, *J* = 9.0 Hz, 2H), 1.88 (t, *J* = 9.0 Hz, 1H), 1.39 (s, 9H). HRMS (ESI), m/z calcd for C_6_H_14_NOS [M+H]^+^ 148.0791, found 148.0795.

***N*-(4-Fluorophenyl)-2-mercaptoacetamide (19h)** was prepared according to General Procedure 3 from **18h** (510 mg, 1.19 mmol), TFA (665 mg, 5.9 mmol) and TES (271 mg, 2.3 mmol). Yield 180 mg, 82 %. ^1^H NMR (400 MHz, CDCl_3_) δ 8.50 (s, 1H), 7.59 – 7.50 (m, 2H), 7.07 (m, 2H), 3.43 (d, *J* = 9.2 Hz, 2H), 2.04 (t, *J* = 9.3 Hz, 1H). ^13^C NMR (126 MHz, CDCl_3_) δ 167.0, 159.7 (d, *J* = 244.2 Hz), 133.3 (d, *J* = 3.0 Hz), 121.7 (d, *J* = 7.9 Hz), 115.8 (d, *J* = 22.6 Hz), 29.0. HRMS (ESI), m/z calcd for C_8_H_9_FNOS [M+H]^+^ 186.0383, found 186.0386.

**2-Mercapto-*N*-(4-methoxyphenyl)acetamide (19i)** was prepared according to General Procedure 3 from **18i** (513 mg, 1.2 mmol), TFA (665 mg, 5.9 mmol) and TES (271 mg, 2.3 mmol). Yield 225 g, 98 %. ^1^H NMR (400 MHz, CDCl_3_) δ 8.41 (s, 1H), 7.50 – 7.41 (m, 2H), 7.02 – 6.78 (m, 2H), 3.82 (s, 3H), 3.41 (d, *J* = 9.2 Hz, 2H), 2.03 (t, *J* = 9.2 Hz, 1H). ^13^C NMR (101 MHz, CDCl_3_) δ 166.9, 156.8, 130.4, 121.7, 114.3, 55.5, 29.0. HRMS (ESI), m/z calcd for C_9_H_12_NO_2_S [M+H]^+^ 198.0583, found 198.0581.

**2-Mercaptoacetamide (19j)** was prepared according to General Procedure 3 from **8j** (708 mg, 2.1 mmol), TFA (1.2 g, 10 mmol) and TES (492 mg, 4.2 mmol). Yield 145 mg, 75 %. ^1^H NMR (400 MHz, CDCl_3_) δ 6.97 (s, 2H), 3.32 (d, *J* = 9.0 Hz, 2H), 2.00 (t, *J* = 9.2 Hz, 1H). ^13^C NMR (101 MHz, CDCl_3_) δ 174.7, 27.8. HRMS (ESI), m/z calcd for C_2_H_6_NOS [M+H]^+^ 92.0165, found 92.0170.

***N*-(Furan-2-ylmethyl)-2-mercaptoacetamide (19k)** was prepared according to General Procedure 3 from **18k** (1g, 2.4 mmol), TFA (1.4 g, 12.1 mmol) and TES (557 mg, 4.8 mmol). Yield 218 mg, 53 % (85% NMR purity). ^1^H NMR (400 MHz, CDCl_3_) δ 7.40 (dd, *J* = 1.9, 0.9 Hz, 1H), 6.98 (s, 1H), 6.36 (dd, *J* = 3.3, 1.9 Hz, 1H), 6.31 – 6.26 (m, 1H), 4.50 (d, *J* = 5.6 Hz, 2H), 3.29 (d, *J* = 9.1 Hz, 2H), 1.90 (t, *J* = 9.1 Hz, 1H). HRMS (ESI), m/z calcd for C_7_H_10_NO_2_S [M+H]^+^ 172.0427, found 172.0432.

**2,2'-Disulfanediylbis(1-(pyrrolidin-1-yl)ethan-1-one) (11a)** was prepared according to General Procedure 4 from **19a** (90 mg, 0.31 mmol) and TEA (66 mg, 0.93 mmol). Yield 66 mg, 75%. ^1^H NMR (400 MHz, CDCl_3_) δ 3.69 (s, 4H), 3.57 (t, *J* = 6.8 Hz, 4H), 3.51 (t, *J* = 6.9 Hz, 4H), 2.04 – 1.96 (m, 4H), 1.95 – 1.84 (m, 4H). ^13^C NMR (126 MHz, CDCl_3_) δ 166.7, 47.2, 46.2, 42.3, 26.2, 24.4. HRMS (ESI), m/z calcd for C_12_H_20_N_2_O_2_S_2_Na [M+Na]^+^ 311.0858, found 311.0873.

**2,2'-Disulfanediylbis(*N*-propylacetamide) (11b)** was prepared according to General Procedure 4 from **19b** (84 mg, 0.63 mmol) and TEA (102 mg, 1 mmol). Yield 45 mg, 54%. ^1^H NMR (400 MHz, CDCl_3_) δ 6.57 (s, 2H), 3.46 (s, 4H), 3.31 (td, *J* = 7.2, 5.9 Hz, 4H), 1.66 – 1.57 (h, *J* = 7.4 Hz, 4H), 0.98 (t, *J* = 7.4 Hz, 6H). ^13^C NMR (101 MHz, CDCl_3_) δ 168.2, 42.7, 41.8, 22.8, 11.4. HRMS (ESI), m/z calcd for C_10_H_20_N_2_O_2_S_2_ [M+Na]^+^ 287.0858, found 287.0865.

**2,2'-Disulfanediylbis(1-morpholinoethan-1-one) (11c)** was prepared according to General Procedure 4 from **19c** (149 mg, 0.9 mmol) and TEA (141 mg, 1.4 mmol). Yield 86 mg, 58%. ^1^H NMR (400 MHz, CDCl_3_) δ 3.75 (d, *J* = 4.5 Hz, 4H), 3.73 (s, 4H), 3.71 (d, *J* = 5.2 Hz, 4H), 3.68 – 3.62 (m, 4H), 3.56 (t, *J* = 4.8 Hz, 4H). ^13^C NMR (101 MHz, CDCl_3_) δ 166.6, 66.7, 66.7, 46.8, 42.4, 40.3. HRMS (ESI), m/z calcd for C_12_H_20_N_2_O_4_S_2_Na [M+Na]^+^ 343.0757, found 343.0770.

**2,2'-Disulfanediylbis(*N*-cyclopropylacetamide) (11d)** was prepared according to General Procedure 4 from **19d** (77 mg, 0.59 mmol) and TEA (122 mg, 1.2 mmol) . Yield 15 mg, 19%. ^1^H NMR (400 MHz, CDCl_3_) δ 6.65 (s, 2H), 3.42 (s, 4H), 2.81 (tq, *J* = 7.3, 3.7 Hz, 2H), 0.84 (td, *J* = 7.1, 5.3 Hz, 4H), 0.66 – 0.60 (m, 4H). ^13^C NMR (126 MHz, CDCl_3_) δ 169.7, 42.5, 23.2, 6.6. HRMS (ESI), m/z calcd for C_10_H_16_N_2_O_2_S_2_Na [M+Na]^+^ 283.0545, found 283.0534.

**2,2'-Disulfanediylbis(*N*-(4-methoxybenzyl)acetamide) (11e)** was prepared according to General Procedure 4 from **19e** (80 mg, 0.38 mmol) and TEA (58 mg, 0.57 mmol). Yield 62 mg, 78%. ^1^H NMR (400 MHz, DMSO-*d6*) δ 8.48 (t, *J* = 5.9 Hz, 2H), 7.24 – 7.14 (m, 4H), 6.97 – 6.79 (m, 4H), 4.22 (d, *J* = 5.8 Hz, 4H), 3.72 (s, 6H), 3.51 (s, 4H). ^13^C NMR (101 MHz, DMSO-*d6*) δ 168.1, 158.7, 131.4, 129.1, 114.2, 55.5, 46.3, 42.5, 42.4. HRMS (ESI), m/z calcd for C_20_H_24_N_2_O_4_S_2_Na [M+Na]^+^ 443.1070, found 443.1090.

**2,2'-Disulfanediylbis(1-(4-methylpiperazin-1-yl)ethan-1-one) (11f)** was prepared according to General Procedure 4 from **19f** (75 mg, 0.43 mmol) and TEA (65 mg, 0.65 mmol). Yield 20 mg, 52%. ^1^H NMR (400 MHz, CDCl_3_) δ 3.75 (s, 4H), 3.67 (t, *J* = 5.2 Hz, 4H), 3.62 – 3.52 (m, 4H), 2.47 (t, *J* = 5.1 Hz, 4H), 2.42 (t, *J* = 5.2 Hz, 4H), 2.34 (s, 6H). ^13^C NMR (101 MHz, CDCl_3_) δ 166.5, 55.1, 54.6, 46.4, 46.0, 42.0, 40.9. HRMS (ESI), m/z calcd for C_14_H_26_N_4_O_2_S_2_Na [M+Na]^+^ 369.1389, found 369.1392.

**2,2'-Disulfanediylbis(*N-(tert*-butyl)acetamide) (11g)** was prepared according to General Procedure 4 from **19g** (70 mg, 0.45 mmol) and TEA (72 mg, 0.71 mmol). Yield 45 mg, 64%. ^1^H NMR (400 MHz, CDCl_3_) δ 6.32 (s, 2H), 3.39 (s, 4H), 1.41 (s, 18H). ^13^C NMR (101 MHz, CDCl_3_) δ 167.3, 51.9, 43.7, 28.7. HRMS (ESI), m/z calcd for C_12_H_24_N_2_O_2_S_2_Na [M+Na]^+^ 315.1171, found 315.1168.

**2,2'-disulfanediylbis(*N*-(4-fluorophenyl)acetamide) (11h)** was prepared according to General Procedure 4 from **18h** (80 mg, 0.43 mmol) and TEA (65 mg, 0.64 mmol). Yield 40 mg, 50%. ^1^H NMR (400 MHz, DMSO-*d*_6_+CDCl_3_) δ 9.72 (s, 2H), 7.67 – 7.45 (m, 4H), 6.93 (m, 4H), 3.58 (s, 4H). ^13^C NMR (101 MHz, DMSO-*d*_6_+CDCl_3_) δ 167.3, 159.2 (d, *J_CF_* = 243.1 Hz), 134.4 (d, *J_CF_* = 2.8 Hz), 121.6 (d, *J_CF_* = 7.9 Hz), 115.4 (d, *J_CF_* = 22.4 Hz), 43.8. HRMS (ESI), m/z calcd for C_16_H_14_F_2_N_2_O_2_S_2_Na [M+Na]^+^ 391.0357, found 391.0352.

**2,2'-disulfanediylbis(*N*-(4-methoxyphenyl)acetamide) (11i)** was prepared according to General Procedure 4 from **19i** (80 mg, 0.41 mmol) and TEA (61 mg, 0.6 mmol). Yield 63 mg, 79 %. ^1^H NMR (400 MHz, DMSO-*d*_6_+CDCl_3_) δ 9.68 (s, 2H), 7.55 – 7.43 (m, 4H), 6.92 – 6.66 (m, 4H), 3.71 (s, 6H), 3.58 (s, 4H). ^13^C NMR (101 MHz, DMSO-*d*_6_+CDCl_3_) δ 167.0, 156.2, 131.6, 121.6, 113.9, 55.4, 43.8. HRMS (ESI), m/z calcd for C_18_H_20_N_2_O_4_S_2_Na [M+Na]^+^ 415.0757, found 415.0759.

**2,2'-Disulfanediyldiacetamide (11j)** was prepared according to General Procedure 4 from **19j** (73 mg, 0.8 mmol) and TEA (202 mg, 2 mmol). Yield 70 mg, 97 %. ^1^H NMR (400 MHz, DMSO-*d*_6_) δ 7.51 (s, 1H), 7.15 (s, 1H), 3.47 (s, 2H). ^13^C NMR (101 MHz, DMSO-*d*_6_) δ 170.3, 42.5. HRMS (ESI), m/z calcd for C_4_H_8_N_2_O_2_S_2_Na [M+Na]^+^ 202.9919, found 202.9924.

**2,2'-Disulfanediylbis(*N*-(furan-2-ylmethyl)acetamide) (4k)** was prepared according to General Procedure 4 from **19k** (84 mg, 0.5 mmol) and TEA (126 mg, 1.25 mmol). Yield 66 mg, 80%. ^1^H NMR (400 MHz, CDCl_3_) δ 7.44 – 7.33 (m, 2H), 6.92 (s, 2H), 6.34 (dd, *J* = 3.2, 1.9 Hz, 2H), 6.29 (d, *J* = 3.2 Hz, 2H), 4.49 (d, *J* = 5.6 Hz, 4H), 3.45 (s, 4H). ^13^C NMR (126 MHz, CDCl_3_) δ 168.3, 150.9, 142.3, 110.5, 107.8, 42.5, 36.9. HRMS (ESI), m/z calcd for C_14_H_16_N_2_O_4_S_2_Na [M+Na]^+^ 363.0444, found 363.0445.

**2-((2-morpholino-2-oxoethyl)disulfanyl)-*N*-propylacetamide (12a)** was prepared according to General Procedure 5 from **19c** (84 mg, 0.63 mmol) as the first thiol, **19b** (102 mg, 0.63 mmol) as the second thiol, BtCl (106 mg, 0.69 mmol) and BtH (75 mg, 0.63 mmol). Yield 70 mg, 38 %. ^1^H NMR (400 MHz, CDCl_3_) δ 3.73 (dt, *J* = 6.4, 3.8 Hz, 4H), 3.69 (d, *J* = 6.2 Hz, 2H), 3.67 (s, 2H), 3.53 (t, *J* = 4.9 Hz, 2H), 3.50 (s, 2H), 3.35 – 3.26 (m, 2H), 1.62 (h, *J* = 7.4 Hz, 2H), 0.98 (t, *J* = 7.4 Hz, 3H). ^13^C NMR (126 MHz, CDCl_3_) δ 167.8, 167.0, 66.8, 66.6, 46.8, 42.9, 42.5, 41.8, 39.9, 22.8, 11.5. HRMS (ESI), m/z calcd for C_11_H_20_N_2_O_3_S_2_Na [M+Na]^+^ 315.0808, found 315.0815.

**Methyl 2-((2-((4-fluorophenyl)amino)-2-oxoethyl)disulfanyl)acetate (13a)** was prepared according to General Procedure 5 from **19h** (110 mg, 0.59 mmol) as the first thiol, methyl thioglycoate (69 mg, 0.65 mmol) as the second thiol, BtCl (100 mg, 0.65 mmol) and BtH (71 mg, 0.6 mmol). Yield 68 mg, 40 %. ^1^H NMR (400 MHz, CDCl_3_) δ 8.71 (s, 1H), 7.67 – 7.43 (m, 2H), 7.05 (m, 2H), 3.84 (s, 3H), 3.65 (s, 2H), 3.60 (s, 2H). ^13^C NMR (101 MHz, CDCl_3_) δ 171.31, 166.03, 159.51 (d, *J_CF_* = 243.7 Hz), 133.79 (d, *J_CF_* = 2.9 Hz), 121.69 (d, *J_CF_* = 7.8 Hz), 115.63 (d, *J_CF_* = 22.5 Hz), 53.17, 42.85, 41.87. HRMS (ESI), m/z calcd for C_11_H_12_FNO_3_S_2_Na [M+Na]^+^ 312.0135, found 312.0139.

**^1^H and ^13^C spectra of compound 18a**

**^1^H and ^13^C spectra of compound 18b**

**^1^H and ^13^C spectra of compound 18c**

**^1^H and ^13^C spectra of compound 18d**

**^1^H and ^13^C spectra of compound 18e**

**^1^H and ^13^C spectra of compound 18f**

**^1^H and ^13^C spectra of compound 11g**

**^1^H and ^13^C spectra of compound 18h**

**^1^H and ^13^C spectra of compound 18i**

**^1^H and ^13^C spectra of compound 18j**

**^1^H and ^13^C spectra of compound 18k**

**^1^H and ^13^C spectra of compound 19a**

**^1^H and ^13^C spectra of compound 19b**

**^1^H and ^13^C spectra of compound 19c**

**^1^H and ^13^C spectra of compound 19e**

**^1^H and ^13^C spectra of compound 19h**

**^1^H and ^13^C spectra of compound 19i**

**^1^H and ^13^C spectra of compound 19j**

**^1^H and ^13^C spectra of compound 11a**

**^1^H and ^13^C spectra of compound 11b**

**^1^H and ^13^C spectra of compound 11c**

**^1^H and ^13^C spectra of compound 11d**

**^1^H and ^13^C spectra of compound 4e**

**^1^H and ^13^C spectra of compound 4f**

**^1^H and ^13^C spectra of compound 11g**

**^1^H and ^13^C spectra of compound 11h**

**^1^H and ^13^C spectra of compound 11i**

**^1^H and ^13^C spectra of compound 11j**

**^1^H and ^13^C spectra of compound 11k**

**^1^H and ^13^C spectra of compound 12a**

**^1^H and ^13^C spectra of compound 13a**
